# Supplementary figures and images for: Optimizing metaproteomics database construction: lessons from a study of the vaginal microbiome
Source: mSystems. 2023 Jun 23;8(4):e00678-22. doi: 10.1128/msystems.00678-22 (PMC10469846; doi:10.1128/msystems.00678-22)

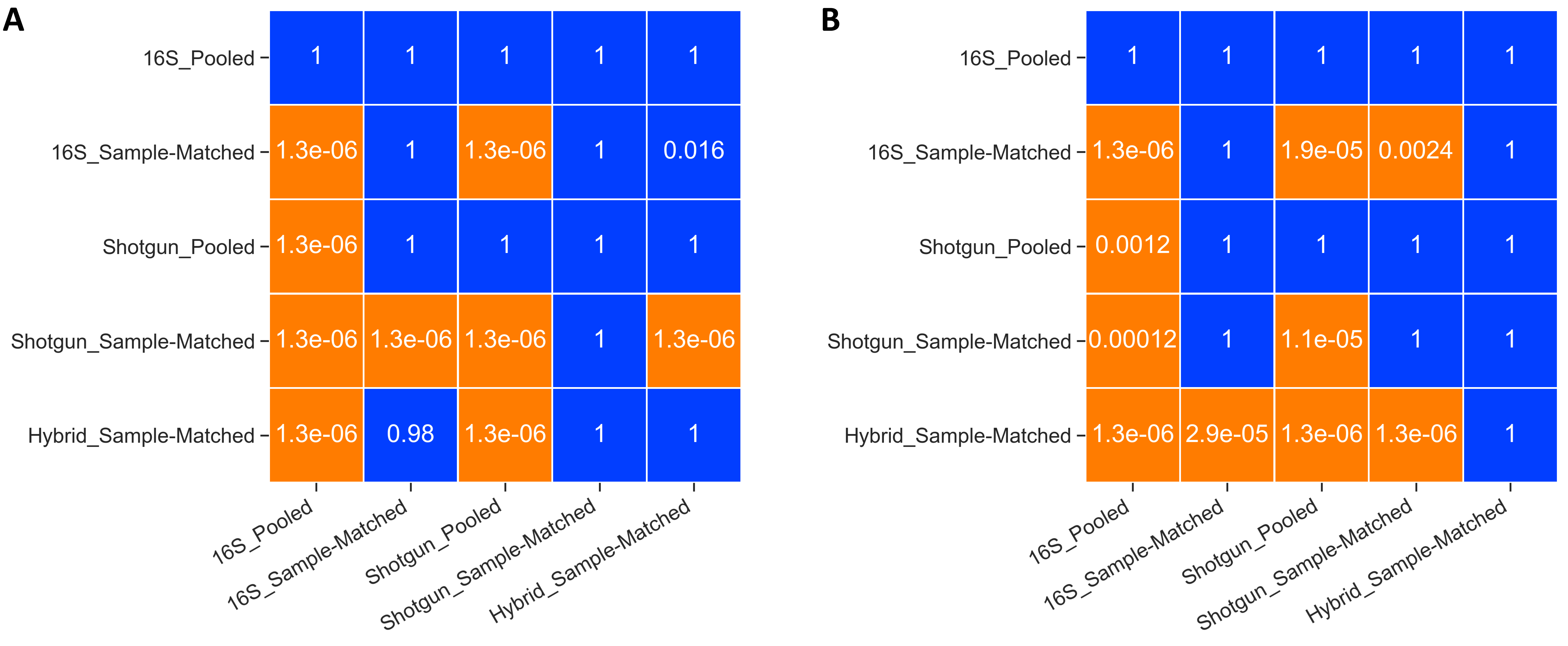

Supplement: Fig. S4 — Comparison of database performance across all samples. All 29 CVL samples were searched against the five listed database types and one-sided Wilcoxon signed-rank tests were performed to determine whether the database listed on the row generated significantly more human (A) or bacterial (B) PSMs than the database listed under the column. The P-value for each test is shown in the cell. Comparisons that were significant (P < 0.01) are shaded orange, while nonsignificant comparisons are shaded blue. [file msystems.00678-22-s0004.tif]
