# Supplementary material for: Optimizing metaproteomics database construction: lessons from a study of the vaginal microbiome
Source: mSystems. 2023 Jun 23;8(4):e00678-22. doi: 10.1128/msystems.00678-22 (PMC10469846; doi:10.1128/msystems.00678-22)
Supplement: Table S1 — Bacterial read counts and alpha diversity from 16S rRNA gene sequencing and shotgun metagenomic sequencing. Number of reads of bacterial DNA and alpha diversity as measured by Shannon index of each sample by both 16S rRNA gene sequencing and shotgun metagenomic sequencing. Shannon index of zero indicates a sample where only one species was detected. [file msystems.00678-22-s0006.docx]

|  | Bacterial Read Count | | Shannon Diversity | |
| --- | --- | --- | --- | --- |
| Sample | 16S | Shotgun | 16S | Shotgun |
| BV-_1 | 23236 | 882434 | 0.487545 | 0.667453 |
| BV-_2 | 20871 | 779090 | 0.053866 | 0.0278757 |
| BV-_3 | 15190 | 2022634 | 0.012724 | 0.0045945 |
| BV-_4 | 15284 | 2900676 | 0.667707 | 1.0015145 |
| BV-_5 | 52505 | 586830 | 0.268073 | 0.6559788 |
| BV-_6 | 51360 | 2026721 | 0.01339 | 0.0185947 |
| BV-_7 | 29925 | 2237111 | 0.167799 | 0.4480193 |
| BV-_8 | 29662 | 490594 | 0.341044 | 0.2372794 |
| BV-_9 | 23458 | 1188354 | 0 | 0 |
| BV+_1 | 35686 | 25276935 | 2.524418 | 1.4765659 |
| BV+_2 | 26968 | 28006006 | 2.377494 | 1.6659287 |
| BV+_3 | 23085 | 21125060 | 2.120943 | 1.5609515 |
| BV+_4 | 22893 | 20962956 | 2.13367 | 1.2927183 |
| BV+_5 | 31317 | 21491262 | 2.089727 | 1.6873654 |
| BV+_6 | 23197 | 727907 | 1.268334 | 1.0887352 |
| BV+_7 | 12921 | 19521942 | 1.790447 | 1.4002334 |
| BV+_8 | 29972 | 18711027 | 1.432482 | 0.8219423 |
| BV+_9 | 23674 | 17722886 | 1.913051 | 1.0760732 |
| BV+_10 | 22442 | 33314551 | 1.675475 | 1.5480884 |
| BV+_11 | 20972 | 14416103 | 2.930262 | 1.9941209 |
| BV+_12 | 39669 | 4067329 | 1.988535 | 1.2449799 |
| BV+_13 | 41211 | 2373332 | 1.62566 | 0.9656578 |
| BV+_14 | 35470 | 10814484 | 1.842138 | 1.0581705 |
| BV+_15 | 51544 | 11189776 | 1.447749 | 1.0805937 |
| BV+_16 | 25693 | 15653291 | 2.58609 | 1.5005845 |
| BV+_17 | 24024 | 12683032 | 2.513713 | 1.5057362 |
| BV+_18 | 20127 | 3635398 | 2.631437 | 2.0558514 |
| BV+_19 | 32622 | 17393941 | 2.153694 | 1.2361104 |
| BV+_20 | 23916 | 8574310 | 2.57824 | 2.066361 |
