# Supplementary material for: Optimizing metaproteomics database construction: lessons from a study of the vaginal microbiome
Source: mSystems. 2023 Jun 23;8(4):e00678-22. doi: 10.1128/msystems.00678-22 (PMC10469846; doi:10.1128/msystems.00678-22)
Supplement: Table S2 — Number of significant PSMs per sample identified by searches of different protein databases. Number of significant human and bacterial PSMs generated in each sample by database type. [file msystems.00678-22-s0007.docx]

| Sample ID | 16S_Pooled | | 16S_Sample-Matched | | | Shotgun_Pooled | | | Shotgun_Sample-Matched | | | Hybrid_Sample-Matched | | |
| --- | --- | --- | --- | --- | --- | --- | --- | --- | --- | --- | --- | --- | --- | --- |
|  | Human | Bacterial | Human | Bacterial | Human | | Bacterial | Human | | Bacterial | Human | | Bacterial |  |
| BV-_1 | 9751 | 73 | 12007 | 127 | 10152 | | 93 | 12198 | | 93 | 12005 | | 126 |  |
| BV-_2 | 7576 | 67 | 9789 | 132 | 8029 | | 95 | 10184 | | 124 | 9789 | | 133 |  |
| BV-_3 | 7964 | 65 | 9851 | 104 | 8277 | | 78 | 10039 | | 79 | 9850 | | 104 |  |
| BV-_4 | 6775 | 168 | 8662 | 352 | 7292 | | 293 | 8949 | | 336 | 8663 | | 366 |  |
| BV-_5 | 8395 | 191 | 10364 | 322 | 8764 | | 269 | 10863 | | 310 | 10363 | | 321 |  |
| BV-_6 | 6085 | 158 | 7983 | 314 | 6492 | | 237 | 8031 | | 303 | 7992 | | 313 |  |
| BV-_7 | 7935 | 63 | 9724 | 160 | 8279 | | 144 | 9889 | | 171 | 9699 | | 179 |  |
| BV-_8 | 7596 | 114 | 9237 | 187 | 8080 | | 129 | 9779 | | 159 | 9236 | | 186 |  |
| BV-_9 | 6907 | 91 | 9051 | 210 | 7148 | | 156 | 9101 | | 202 | 9052 | | 211 |  |
| BV+_1 | 4949 | 874 | 6128 | 1161 | 5287 | | 872 | 6308 | | 909 | 6066 | | 1172 |  |
| BV+_2 | 1688 | 1667 | 2228 | 2494 | 1879 | | 2154 | 2401 | | 2319 | 2239 | | 2915 |  |
| BV+_3 | 4128 | 3691 | 5316 | 4817 | 4526 | | 3530 | 5473 | | 3599 | 5271 | | 4849 |  |
| BV+_4 | 3258 | 1387 | 4314 | 2028 | 3553 | | 1754 | 4411 | | 1841 | 4293 | | 2410 |  |
| BV+_5 | 4547 | 169 | 5733 | 265 | 4750 | | 176 | 5904 | | 212 | 5772 | | 280 |  |
| BV+_6 | 4413 | 579 | 5887 | 889 | 4985 | | 1050 | 6216 | | 1215 | 5920 | | 1286 |  |
| BV+_7 | 8688 | 485 | 10292 | 597 | 8944 | | 421 | 10585 | | 422 | 10294 | | 598 |  |
| BV+_8 | 6560 | 298 | 8271 | 458 | 6871 | | 296 | 8388 | | 344 | 8190 | | 459 |  |
| BV+_9 | 6196 | 1085 | 7669 | 1452 | 6561 | | 1072 | 7909 | | 1180 | 7621 | | 1500 |  |
| BV+_10 | 6839 | 197 | 8496 | 392 | 7092 | | 298 | 8580 | | 350 | 8462 | | 390 |  |
| BV+_11 | 5645 | 280 | 6874 | 399 | 6031 | | 404 | 7036 | | 460 | 6884 | | 537 |  |
| BV+_12 | 4727 | 353 | 6011 | 500 | 4844 | | 324 | 6300 | | 356 | 5992 | | 509 |  |
| BV+_13 | 5812 | 654 | 7489 | 913 | 6254 | | 658 | 7811 | | 635 | 7446 | | 986 |  |
| BV+_14 | 6637 | 982 | 8325 | 1318 | 7301 | | 1025 | 8508 | | 998 | 8299 | | 1358 |  |
| BV+_15 | 5152 | 629 | 6480 | 809 | 5544 | | 598 | 6608 | | 570 | 6416 | | 812 |  |
| BV+_16 | 7383 | 177 | 9155 | 275 | 7944 | | 220 | 9401 | | 249 | 9247 | | 315 |  |
| BV+_17 | 5369 | 567 | 6705 | 780 | 5747 | | 768 | 6873 | | 851 | 6656 | | 997 |  |
| BV+_18 | 4909 | 61 | 6135 | 87 | 5056 | | 80 | 6379 | | 115 | 6108 | | 130 |  |
| BV+_19 | 4215 | 186 | 5246 | 282 | 4402 | | 185 | 5331 | | 193 | 5235 | | 296 |  |
| BV+_20 | 5391 | 319 | 6892 | 469 | 5785 | | 521 | 7120 | | 565 | 6889 | | 674 |  |
