# Supplementary material for: Optimizing metaproteomics database construction: lessons from a study of the vaginal microbiome
Source: mSystems. 2023 Jun 23;8(4):e00678-22. doi: 10.1128/msystems.00678-22 (PMC10469846; doi:10.1128/msystems.00678-22)
Supplement: Table S3 — Percentage of significant PSMs per sample identified to specific taxa by searches of different protein databases. Average percent of bacterial PSMs matched to proteins from only one genus or species by searches of the listed database type. [file msystems.00678-22-s0008.docx]

| **Database** | **Genus** | **Species** |
| --- | --- | --- |
| **16S_Pooled** | **86.0%** | **42.9%** |
| **16S_Sample-Matched** | **95.8%** | **50.1%** |
| **Shotgun_Pooled** | **48.9%** | **46.3%** |
| **Shotgun_Sample-Matched** | **50.0%** | **49.4%** |
| **Hybrid_Sample-Matched** | **89.1%** | **43.8%** |
