# Supplementary material for: Optimizing metaproteomics database construction: lessons from a study of the vaginal microbiome
Source: mSystems. 2023 Jun 23;8(4):e00678-22. doi: 10.1128/msystems.00678-22 (PMC10469846; doi:10.1128/msystems.00678-22)
Supplement: Table S4 — Taxa associated with spectra identified in 16S_Sample-Matched database search and missed by Shotgun_Sample-Matched search, and vice-versa. The number of spectra across all samples that were matched to each taxonomic identifier and were identified by the 16S_Sample-Matched search and missed by searching the corresponding Shotgun_Sample-Matched search, or vice versa. [file msystems.00678-22-s0009.docx]

| 16S_Sample-Matched | | Shotgun_Sample-Matched | |
| --- | --- | --- | --- |
| Assigned Taxa | Total Spectra | Assigned Taxa | Total Spectra |
| *Gardnerella* | 4929 | *Gardnerella* | 271 |
| *Prevotella timonensis* | 278 | *Prevotella timonensis* | 30 |
| *Prevotella buccalis* | 153 | *Prevotella* | 129 |
| *Fannyhessea vaginae* | 145 | *Fannyhessea vaginae* | 13 |
| *Megasphaera lornae* | 125 | *Megasphaera lornae* | 8 |
| *Prevotella disiens* | 125 | *Prevotella disiens* | 12 |
| *Prevotella bivia* | 117 | *Prevotella bivia* | 8 |
| *Lactobacillus crispatus* | 114 | *Lactobacillus crispatus* | 11 |
| *Lactobacillus iners* | 109 | *Lactobacillus iners* | 30 |
| *Prevotella amnii* | 56 | *Prevotella amnii* | 4 |
| *Porphyromonas uenonis* | 50 | *Porphyromonas* | 4 |
| *Lactobacillus jensenii* | 37 | Bacilli | 3 |
| *Peptoniphilus lacrimalis* | 34 | *Peptoniphilus* | 7 |
| Candidatus *Lachnocurva vaginae* | 31 | *Lachnospiraceae* | 3 |
| *Mobiluncus mulieris* | 30 | *Mobiluncus mulieris* | 15 |
| *Peptostreptococcus anaerobius* | 24 | *Peptostreptococcus anaerobius* | 1 |
| *Dialister micraerophilus* | 21 | *Dialister micraerophilus* | 2 |
| *Megasphaera hutchinsoni* | 18 | *Megasphaera* | 28 |
| *Prevotella bergensis* | 17 | *Prevotella bergensis* | 4 |
| *Mobiluncus curtisii* | 14 | *Mobiluncus curtisii* | 1 |
| *Finegoldia magna* | 13 | Bacteria | 15 |
| *Dialister* sp | 12 | *Dialister* sp. type 2 | 3 |
| *Sneathia vaginalis* | 10 | *Sneathia* | 8 |
| Peptoniphilus grossensis | 10 | Unknown | 1667 |
| *Porphyromonas* sp | 8 | *Clostridiales* bacterium KA00274 | 345 |
| *Parvimonas micra* | 7 | Terrabacteria group | 308 |
| *Prevotella intermedia* | 5 | *Prevotellaceae* | 8 |
| *Arcanobacterium haemolyticum* | 5 | *Arcanobacterium* sp. S3PF19 | 4 |
| *Aerococcus christensenii* | 5 | *Aerococcus christensenii* | 6 |
| *Anaerococcus prevotii* | 5 | *Anaerococcus lactolyticus* | 2 |
| *Mobiluncus holmesii* | 5 | *Mobiluncus* | 11 |
| *Lactobacillus reuteri* | 4 | *Lactobacillus* | 50 |
| *Mageeibacillus indolicus* | 4 | *Mageeibacillus indolicus* | 7 |
| *Sneathia sanguinegens* | 4 | *Tissierellia* | 18 |
| *Sutterella* sp | 3 | *Atopobium* | 10 |
| *Fusobacterium nucleatum* | 3 | *Leptotrichiaceae* | 6 |
| *Ezakiella massiliensis* | 2 | *Veillonellaceae* bacterium DNF00751 | 3 |
| *Prevotella colorans* | 2 | *Actinomycetaceae* | 2 |
| *Bacteroides thetaiotaomicron* | 2 | *Bacteroidales* | 42 |
| *Bacteroides faecis* | 2 | *Bacteroidales* bacterium WCE2008 | 2 |
| *Eggerthella-*like | 2 | *Coriobacteriales* bacterium DNF00809 | 4 |
| *Veillonella atypica* | 1 | *Veillonellaceae* | 46 |
| *Bifidobacterium dentium* | 1 | *Bifidobacteriaceae* | 31 |
| *Campylobacter ureolyticus* | 1 | *Campylobacter* | 1 |
| *Anaerococcus vaginalis* | 1 | *Anaerococcus* | 2 |
| *Megasphaera micronuciformis* | 1 | *Peptostreptococcus* | 2 |
| *Streptococcus agalactiae* | 1 | *Streptococcaceae* | 2 |
| *Sutterella wadsworthensis* | 1 | *Bifidobacterium* | 3 |
| Candidatus TM7 | 1 | *Veillonella* | 2 |
| *Porphyromonas endodontalis* | 1 | *Tissierellia* bacterium KA00581 | 2 |
|  |  | *Gemella asaccharolytica* | 2 |
|  |  | *Atopobiaceae* | 1 |
|  |  | *Tissierellia incertae sedis* | 1 |
|  |  | *Bacteroidia* | 1 |
|  |  | *Coriobacteriales* | 1 |
|  |  | *Lachnospiraceae* bacterium | 1 |
|  |  | *Olsenella* | 1 |
