# Supplementary material for: Optimizing metaproteomics database construction: lessons from a study of the vaginal microbiome
Source: mSystems. 2023 Jun 23;8(4):e00678-22. doi: 10.1128/msystems.00678-22 (PMC10469846; doi:10.1128/msystems.00678-22)
Supplement: Table S5 — Comparison of present techniques with past investigations of the vaginal metaproteome in terms of identified human and bacterial proteins, samples analyzed, and database type. Comparison of the number of unique proteins identified in studies of the vaginal metaproteome, past investigations, and the current study. a. Number of samples analyzed. b. The study did not report the number of this type of proteins identified. [file msystems.00678-22-s0010.docx]

| **Publication** | ^a^***N*** | **Database Type** | **Human** | **Bacterial** |
| --- | --- | --- | --- | --- |
| Dasari, *et al.* 2007 (70) | 7 | Swissprot Human | 150 | ^b^N/A |
| Shaw, *et al.* 2007 (71) | 2 | IPI Human | 685 | N/A |
| Zegels, *et al.* 2009 (72) | 6 | Swissprot Human | 339 | N/A |
| Burgener, *et al.* 2011 (73) | 293 | IPI Human | 360 | N/A |
| Birse, *et al.* 2015 (74) | 19 | Swissprot Human + Swissprot Bacteria | 384 | N/A |
| Muytjens, *et al.* 2017 (75) | 10 | Swissprot Human | 1087 | N/A |
| Ferreira, *et al.* 2018 (38) | 58 | Uniprot Human | 74 | N/A |
| Starodubtseva, *et al.* 2019 (76) | 73 | Swissprot | 675 | N/A |
| Kumar, *et al.* 2021 (77) | 60 | Uniprot Human | 1015 | N/A |
| Klatt, *et al.* 2017 (78) | 688 | Swissprot Human + Two-step Uniprot Bacteria | N/A | 3334 |
| Cruciani, *et al.* 2013 (79) | 80 | Swissprot | 118 | 13 |
| Arnold, *et al.* 2014 (80) | 36 | Database Not Specified | 650 | 100 |
| Borgdorff, *et al.* 2016 (81) | 50 | Swissprot Human + NCBI Vaginal Lactobacilli + NCBI Vaginal Microbes | 549 | 40 |
| Zevin, *et al.* 2016 (37) | 10 | Swissprot Human + Uniprot Bacteria | 434 | 689 |
| Bradley, *et al.* 2018 (39) | 16 | Swissprot Human + Two-step Uniprot Bacteria | 406 | 106 |
| Farr Zuend, *et al.* 2020 (82) | 48 | Swissprot Human + Two-step Uniprot Bacteria | 550 | 376 |
| Alisoltani, *et al.* 2020 (83) | 113 | Uniprot Human + Uniprot Microbes | 1236 | 1778 |
| Nunn, *et al.* 2020 (49) | 4 | Uniprot Human + Translated Sample Metagenomes | 3334 | 1092 |
| 16S_Sample-Matched Databases (this study) | 29 | Two-step, Sample-matched Swissprot Human + NCBI Bacteria present by 16S Seq | 1072 | 1257 |
| Shotgun_Sample-Matched Databases (this study) | 29 | Two-step, Sample-matched Swissprot Human + Translated Sample Metagenomes | 1182 | 942 |
| Hybrid_Sample-Matched Databases (this study) | 29 | Two-step, Sample-matched Swissprot Human + NCBI Bacteria present by 16S Seq + Sample Metagenomes | 1068 | 1418 |
